# Geschlechterverteilung in der Viszeralchirurgie: 11 Jahre Viszeralmedizin-Kongress – ein Aufruf zu mehr Chancengleichheit

**DOI:** 10.1007/s00104-026-02491-4

**Published:** 2026-03-25

**Authors:** Marika Ebner, Marie-Louise Witte, Jennifer Gill, Lea Pueschel, Heiner Wedemeyer, Henrike Lenzen, Katja Schlosser, Miriam Wiestler

**Affiliations:** 1 Unabhängige Wissenschaftlerin, Lübeck, Deutschland; 2 Institut für Angewandte Datenwissenschaft Hannover DATA|H, Hochschule Hannover (HsH), Hannover, Deutschland; 3 Klinik für Innere Medizin, DRK Krankenhaus Clementinenhaus, Hannover, Deutschland; 4 Klinik für Gastroenterologie, Hepatologie, Infektiologie und Endokrinologie, Medizinische Hochschule Hannover, Carl-Neuberg-Str. 1, 30625 Hannover, Deutschland; 5 Klinik für Gastroenterologie, Hepatologie, Interventionelle Endoskopie und Diabetologie, Städtisches Klinikum Braunschweig, Braunschweig, Deutschland; 6 Klinik für Allgemein- und Viszeralchirurgie, Diakovere Krankenhaus Hannover, Hannover, Deutschland

**Keywords:** Geschlechtergerechtigkeit in der Chirurgie, Unterrepräsentation von Frauen, Gläserne Decke, Viszeralmedizin-Kongress, Parität, Gender equality in surgery, Underrepresentation of women, Glass ceiling, Visceral medicine congress, Parity

## Abstract

**Hintergrund und Fragestellung:**

Trotz steigenden Frauenanteils sind Chirurginnen in klinischen und akademischen Führungspositionen weiterhin unterrepräsentiert. Der Viszeralmedizin-Kongress als größter deutschsprachiger Fachkongress ermöglicht die Untersuchung geschlechtsspezifischer Repräsentationsmuster. Ziel der Studie war die Analyse der Geschlechterverteilung in Kongressfunktionen 2013 bis 2024 zur Identifikation möglicher struktureller Barrieren.

**Studiendesign und Untersuchungsmethoden:**

Die Geschlechterverteilung von Vortragenden und Vorsitzenden wurde anhand der Kongress Programme 2013 bis 2024 erfasst. Nach Ausschluss gastroenterologischer, endoskopischer und interdisziplinärer Sitzungen wurden ausschließlich viszeralchirurgische Beiträge ausgewertet.

**Ergebnisse:**

Es zeigte sich ein signifikanter Anstieg weiblicher Vortragender (r^2^ = 0,585; *p* = 0,006) und Vorsitzender (r^2^ = 0,777; *p* < 0,001). Zunahmen fanden sich in Plenarsitzungen (Vortragende r^2^ = 0,600, *p* = 0,005; Vorsitze r^2^ = 0,796, *p* < 0,001) und Abstractsitzungen (Vortragende r^2^ = 0,445, *p* = 0,025; Vorsitze r^2^ = 0,678, *p* = 0,002). Parität wurde nur bei Abstractvorsitzen stellenweise erreicht. In Industriesymposien waren 88–100 % der Vortragenden und 100 % der Vorsitzenden männlich. Von 2013 bis 2024 waren 92 % der Kongresspräsident*innen männlich. Auch bei den Auszeichnungen dominierten Männer: 66,6–80 % der Preisträger und 87,5–100 % der Medaillenempfänger waren männlich.

**Schlussfolgerung:**

Chirurginnen bleiben damit in prestigeträchtigen Kongressfunktionen deutlich unterrepräsentiert und erscheinen überwiegend in weniger sichtbaren Formaten, was strukturelle Barrieren nahelegt. Paritätische Vorgaben führten zu einem signifikanten Anstieg weiblicher Beteiligung. Institutionelle Maßnahmen können die Repräsentation von Frauen in der akademischen Chirurgie verbessern und bestehende Ungleichgewichte reduzieren.

**Graphic abstract:**

**Figure:**
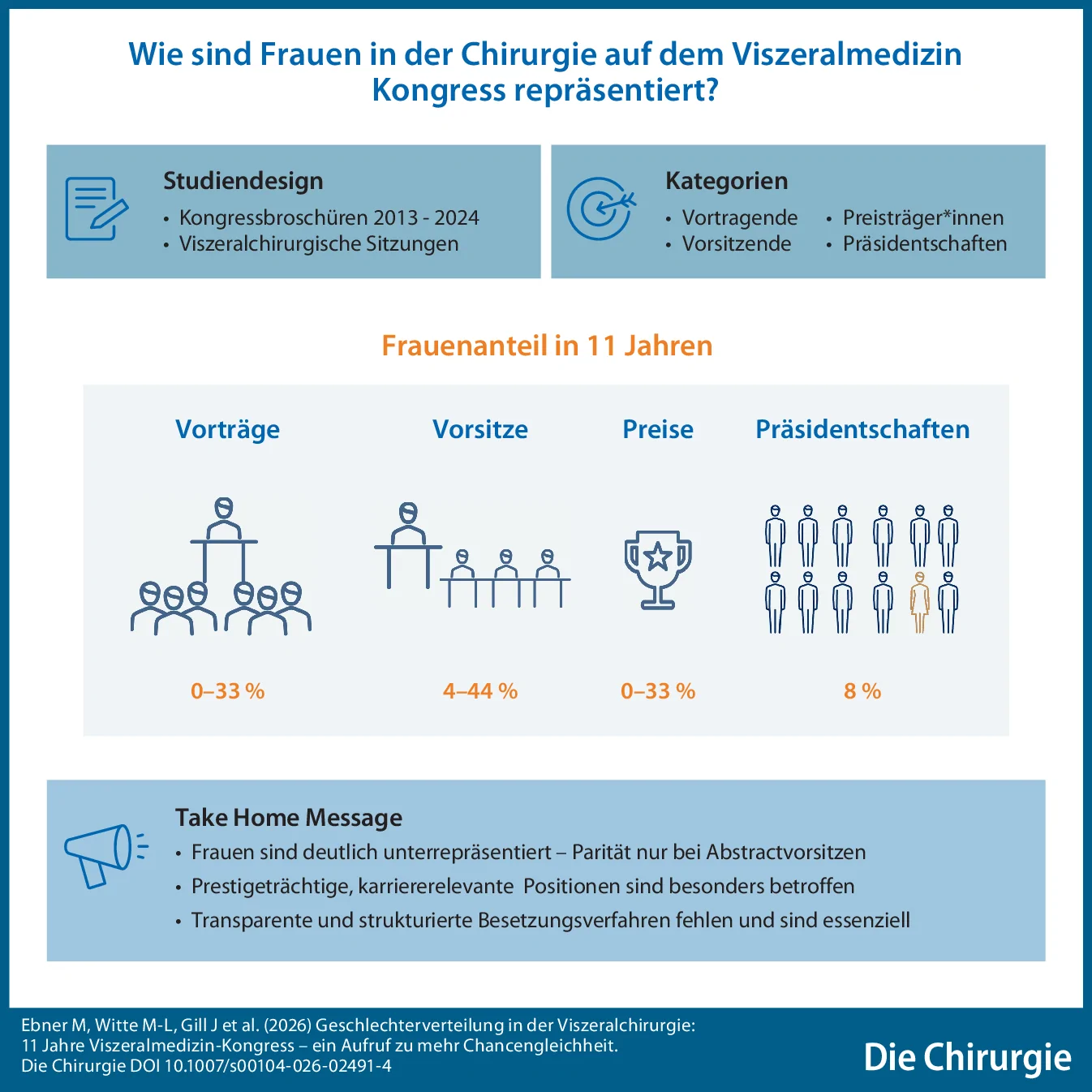

## Abstract (en)

**Background and objective:**

Despite a growing proportion of women in surgery, there is a significant underrepresentation in clinical and academic leadership positions. The mechanisms are still poorly understood. The German Congress of Visceral Medicine, as the largest interdisciplinary medical congress in German-speaking countries, provides a suitable platform for investigating gender-specific representation patterns. The aim of this study was to analyze the gender distribution in various congress functions between 2013 and 2024 in order to identify potential structural inequalities.

**Material and methods:**

The gender distribution of speakers and chairs at the German Congress of Visceral Medicine from 2013 to 2024 was analyzed based on the official congress programs. After excluding gastroenterological, endoscopic and interdisciplinary sessions, the visceral surgery sessions were analyzed in the categories of abstract sessions, plenary sessions and industry symposia.

**Results:**

The analysis revealed a significant increase in the number of female speakers (r^2^ = 0.585; *p* = 0.006) and chairs (r^2^ = 0.777; *p* < 0.001). The proportion of female speakers significantly increased in both plenary sessions (r^2^ = 0.600, *p* = 0.005) and abstract sessions (r^2^ = 0.445, *p* = 0.025). An increase in the number of female chairs was also observed for plenary sessions (r^2^ = 0.796; *p* < 0.001) and abstract sessions (r^2^ = 0.678; *p* = 0.002). Parity could not be achieved except for chairs of abstract sessions. At industry symposia, speakers were male in 88–100%, and chairs were male in 100% of all cases. The congress presidency was held by men in 10 out of 11 years (92%). The Walter Kausch Medal and the Rudolf Pichlmayr Medal went exclusively to men (100%).

**Conclusion:**

The analysis reveals a persistent underrepresentation of female surgeons in prestigious conference roles. Women were predominantly represented in less visible formats, indicating structural barriers. Following the introduction of transparent selection procedures and gender parity requirements, a significant increase in female participation was observed. These results suggest that targeted institutional measures are suitable for promoting the representation of women in academic surgery and reducing existing imbalances.

Frauen bleiben in prestigeträchtigen Funktionen chirurgischer Fachkongresse unterrepräsentiert. Der deutsche Viszeralmedizin-Kongress stellt hierbei keine Ausnahme dar. Dies reduziert Sichtbarkeit, wissenschaftliche Anerkennung und Karrierechancen. Strukturelle Maßnahmen wie paritätische Vorsitzvergabe zeigen erste Wirkung, freiwillige Ansätze reichen jedoch nicht aus. Die vorliegende Analyse untersucht die Geschlechterverteilung auf dem Viszeralmedizin-Kongress 2013 bis 2024 und zeigt Ansatzpunkte zur Förderung von Chancengleichheit auf.

## Hintergrund und Fragestellung

Was kann es zur Gleichstellung der Geschlechter in der Medizin Neues geben? Waren 1908 nur 2–5 % der medizinstudierenden Frauen, stieg ihr Anteil in den 1970er-Jahren auf ein Drittel und liegt heute bei 70 % [8, 30]. Vor 24 Jahren wurde mit Professorin Dr. Henne-Bruns erstmals eine Frau auf einen chirurgischen Lehrstuhl berufen [9]. Allein 2024 erschienen 1195 Publikationen zur Gleichberechtigung in der Medizin [25]. Die Diskussion ist also präsent – in der Chirurgie bleibt gelebte Gleichstellung jedoch weiterhin begrenzt. Im Jahr 2024 waren von 437.162 berufstätigen Ärzt*innen in Deutschland 50 % weiblich, in der Allgemein- und Viszeralchirurgie jedoch nur 36 %. Mit Blick auf klinische und akademische Leitungspositionen reduziert sich der Anteil von Viszeralchirurginnen auf nur 1–5 % [3, 29, 32].

Diese Zahlen verdeutlichen das in der Literatur beschriebene Problem, dass Frauen in der Chirurgie trotz gleicher Qualifikation spezifische Hürden erleben, die ihre Karriereentwicklung behindern und zu vorzeitigem Ausstieg aus der Weiterbildung beitragen [18, 20, 21, 27, 29, 33]. Dadurch bleibt die Zahl von Frauen in Führungspositionen niedrig – die sog. „Gläserne Decke“ [23]. Eine erfolgreiche akademische Laufbahn setzt Publikationen, eingeworbene Forschungsgelder und Vorträge auf Fachkongressen voraus. Einladungen als Vortragende oder Vorsitzende erhöhen Sichtbarkeit und verleihen wissenschaftliche Anerkennung [22]. Kongressbeiträge steigern zudem die Wahrscheinlichkeit einer Veröffentlichung [34]. Ebenso wichtig sind Netzwerke und wissenschaftliche Integration, die maßgeblich zur akademischen Reputation beitragen [6, 23, 26]. Trotz dieser Bedeutung ist der Anteil weiblicher Vortragender auf medizinischen Kongressen weiterhin gering [1, 28]. Die geschlechtsspezifische Diskrepanz kann somit direkt Aufstiegschancen beeinflussen und die Förderung von Frauen in Wissenschaft und Medizin behindern. Der deutsche Viszeralmedizin-Kongress unter der Schirmherrschaft von DGAV und DGVS ist der größte Kongress für Gastroenterologie, Endoskopie und Viszeralchirurgie im deutschsprachigen Raum. Mit jährlich über 6000 Teilnehmenden zählt er zu den größten medizinischen Kongressen [11]. Das umfangreiche Programm umfasst eingeladene Vorträge, die Präsentation eigener Forschung nach entsprechender Bewerbung (Abstracts), die Vergabe von angesehenen Forschungspreisen und von der pharmazeutischen und medizinproduktherstellenden Industrie organisierte Symposien. Letztere sind besonders bedeutsam, da hier früh Informationen über neue Therapien und Medizinprodukte vermittelt werden und Industriekooperationen eine Finanzierungsquelle klinischer Forschung darstellen. Da eingeworbene Fördermittel entscheidend für Beförderungen sind, spielen die hohen Fördersummen der Industrie eine wichtige Rolle für den beruflichen Aufstieg [27]. Es ist allerdings in keinem der genannten Bereiche transparent, wie die Auswahl der Hauptvortragenden erfolgt.

Ziel der vorliegenden Studie war es daher, die Geschlechterverteilung unter Vortragenden, Vorsitzenden, Preisträger*innen und Präsident*innen in der Viszeralchirurgie auf dem Viszeralmedizin-Kongress von 2013 bis 2024 zu analysieren.

## Studiendesign und Untersuchungsmethoden

Diese Untersuchung basiert auf einem Datensatz, der zwischen Dezember 2024 und März 2025 erhoben wurde. Ziel war es, die Geschlechterverteilung auf dem jährlichen deutschen Viszeralmedizin-Kongress von 2013 bis 2024 zu untersuchen, wobei der Schwerpunkt auf Vortragenden und Vorsitzenden lag. Mögliche Unterschiede in der Geschlechterverteilung der viszeralchirurgischen Sitzungen wurden identifiziert und untersucht. Die Untersuchung wurde für alle Sitzungen der Gastroenterologie, Endoskopie und Viszeralchirurgie durchgeführt.

### Erklärung der Deutschen Gesellschaft für Verdauungs- und Stoffwechselkrankheiten zur Parität

Die DGVS hat im Mai 2019 einen Beschluss zur paritätischen Besetzung der Vorsitze verabschiedet. Es war ein Beschluss des DGVS-Vorstandes, der damit auch für die Sektion Endoskopie Wirkung entfaltet. Die DGAV trägt den Beschluss mit.

### Selektionsprozess

Während für Abstractsitzungen eigens initiierte Einreichungen möglich sind, werden Vortragende für die renommierten Plenarsitzungen oder Industriesymposien eingeladen. Es sind keine frei verfügbaren Informationen darüber zugänglich, wie dieser Einladungsprozess abläuft, ob er leistungsbezogen oder in irgendeiner Form standardisiert ist. Ebenso gibt es keine frei verfügbaren Informationen über das Auswahlverfahren für die Vorsitzenden.

### Protokoll

Für den Umgang mit fehlenden Daten, Mehrdeutigkeiten und der geschlechtlichen Zuordnung wurde ein internes Protokoll entwickelt. Forscherin 1 ordnete die in den Kongressprogrammen genannten Personen anhand der Namen einem Geschlecht zu; die Auswertung erfolgte anschließend durch Forscherin 2. Abgekürzte oder nicht eindeutig recherchierbare Namen wurden als „nicht zuordenbar“ klassifiziert. Gleiches galt für Namen, deren Nomenklatur keine eindeutige Zuordnung erlaubte, auch unter Nutzung umfangreicher Online-Suchtools. Die Namenszuordnung stellt keine Bestimmung der Geschlechtsidentität dar; diese ist daher nicht Gegenstand der Analyse. Berücksichtigt wurden ausschließlich Daten aus den Jahren 2013 bis 2024, um Verzerrungen durch externe Entwicklungen wie die Einführung der Frauenquote im deutschen Recht (2016), den Vorstandsbeschluss der DGVS zur paritätischen Besetzung der Vorsitze (2019) oder die SARS-CoV-2-Pandemie (2020–2022) zu minimieren.

### Datenerhebung und -extraktion

Die Kongressbroschüren ab 2013 wurden vollständig heruntergeladen. Da die Nomenklatur für Vortragende und Vorsitzende nicht standardisiert war und teils vollständige Namen, teils Initialen verwendet wurden, erfolgte eine ergänzende Recherche der jeweiligen Affiliationen. Zwischen dem 1. Januar und 1. Februar 2025 ordnete Forscherin 1 die genannten Personen anhand einer umfangreichen Online-Recherche einem Geschlecht zu; für jedes Kongressjahr wurde ein standardisiertes Erhebungsformular verwendet. Forscherin 2 prüfte anschließend die interne Konsistenz des Datensatzes, und Abweichungen wurden im Team diskutiert. Eine Zuordnung erfolgte nur bei Konsens. Bei Unsicherheiten wurden Abstracts oder Beiträge über ResearchGate eingesehen; Fotos dienten ergänzend der Identifikation. Zudem wurde eine Überprüfung geschlechtsspezifischer Namen einschließlich kulturell variierender Vornamen (z. B. „Kim“; „Andrea“) vorgenommen. Institutionelle Webseiten wurden auf geschlechtsspezifische Anreden hin ausgewertet. Fälle, in denen trotz dieser Maßnahmen keine eindeutige Zuordnung möglich war, wurden als „nicht zuordenbar“ klassifiziert und aus der Analyse ausgeschlossen; dies betraf insgesamt 3 Personen (0,07 %).

### Auszeichnungen und Medaillen

Daten zu den Preisträger*innen der DGAV-Auszeichnungen wurden den Kongressbroschüren entnommen und anschließend ausgewertet.

### Zusammenstellung der Daten und konsekutive Analyse

**Abb. 1:**
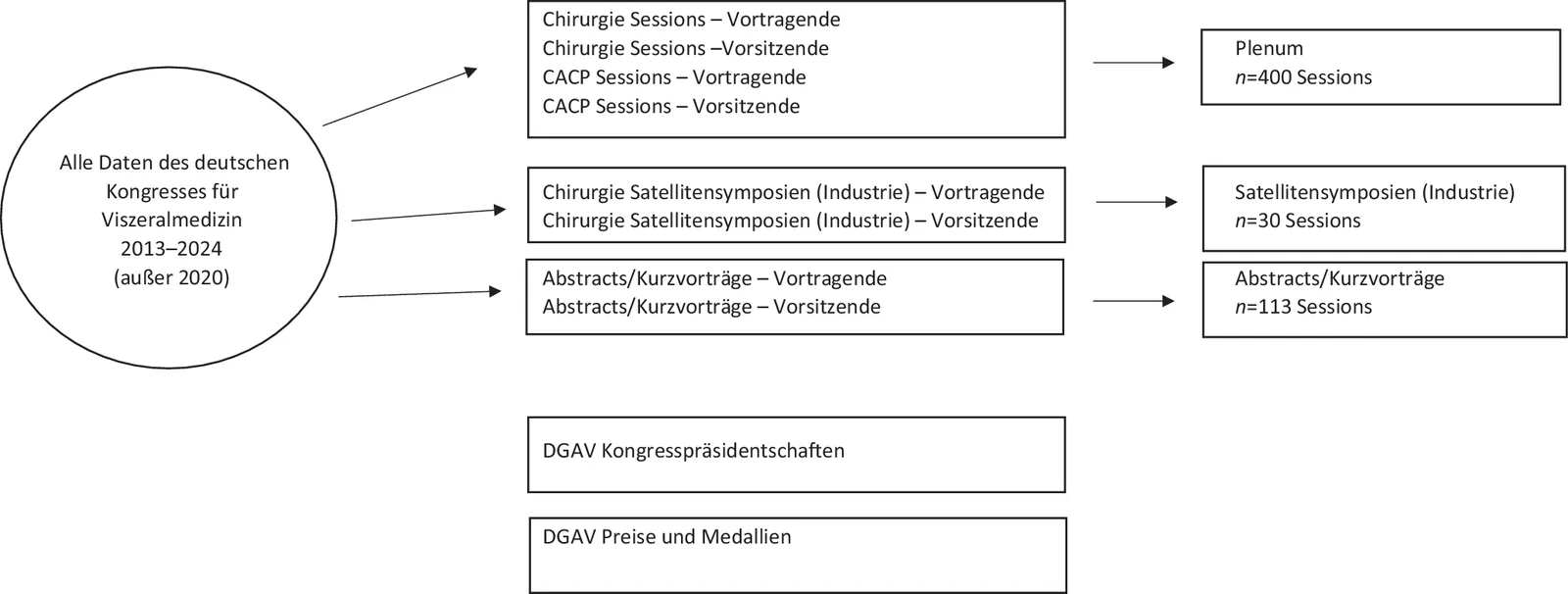
Schritte und Ergebnisse der Datensynthese-Cluster

Die Analyse erfolgte mit Daten der viszeralchirurgischen Sitzungen. Gastroenterologische, endoskopische und interdisziplinäre Sitzungen wurden von dieser Analyse ausgeschlossen und separat ausgewertet. Zunächst wurde der Datensatz gemäß einer methodischen Strukturierung des Programminhalts, angelehnt an die Kongresskonfiguration, in verschiedene Kategorien gegliedert. In der darauffolgenden Phase wurden die Daten weiter unterteilt, wodurch 3 verschiedene Bereiche identifiziert wurden: (1) Plenum, (2) Satellitensymposien (Industrie) und (3) Abstract/Kurzvorträge. Die Bereiche 1–3 wurden anschließend für die Analyse in der Kategorie „Gesamt“ zusammengefasst (Abb. 1). Die Daten aus jedem Bereich bzw. jeder Kategorie wurden jeweils getrennt für Vortragende und Vorsitzende analysiert. Darüber hinaus wurde das Jahr 2020 aufgrund der SARS-CoV-2-Pandemie, die zur Absage von Präsenzkongressen führte, von der Analyse ausgeschlossen.

## Statistik

Die statistische Analyse erfolgte mit der Software SPSS Statistics, Version 28.0.1.0 (SPSS, IBM, Armonk, NY, USA), und GraphPad PRISM, Version 10.3.0 (GraphPad Software, Boston, Massachusetts, USA). Weitere Grafiken wurden mittels Python erstellt. Die lineare Regression diente als primäre Analysemethode zur Trendanalyse des Frauenanteils. Darüber hinaus wurden Cochran-Armitage-Tests zur Trendanalyse eingesetzt. Eine vergleichende Analyse der geschlechtsspezifischen Anzahl der Vorsitzenden im Jahr unmittelbar vor (2018) und nach (2019) der DGVS-Stellungnahme zur Parität erfolgte mit dem Fisher’s Exact-Test. Wichtig zu beachten ist, dass die DGVS-Stellungnahme zur Parität nur für Vorsitzende gilt. Daher wurde diese Analyse nicht für die Daten der Vortragenden durchgeführt.

## Ergebnisse

### Trendanalyse: Frauenanteil unter den Vortragenden (2013–2024)

**Abb. 2:**
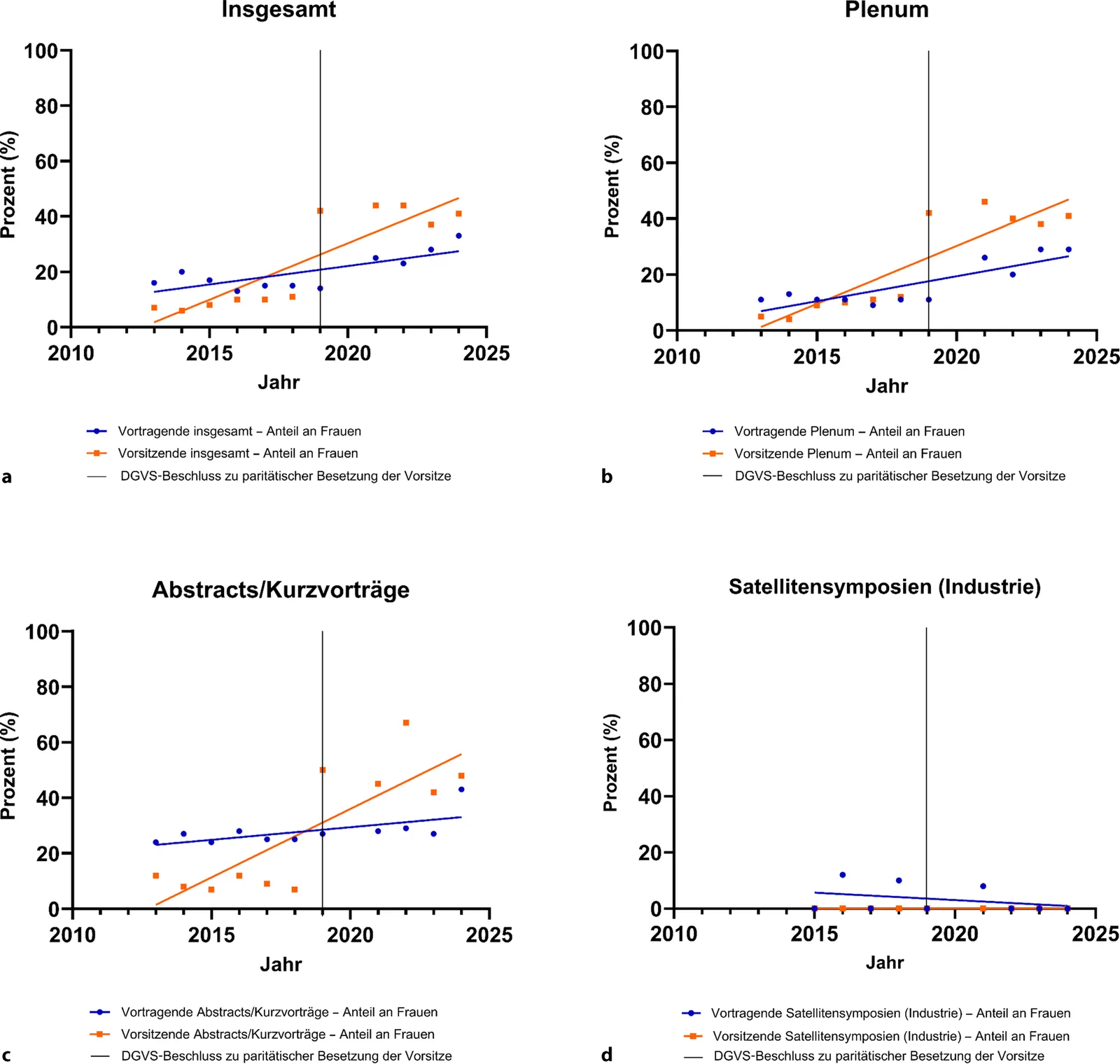
**a–d** Weibliche Vorsitzende insgesamt (**a**), in Plenarsitzungen (**b**), in Abstractsitzungen (**c**) und Satellitensymposien (Industrie) (**d**)

Zwischen 2013 und 2024 gab es bei den Frauenanteilen unter den Vortragenden signifikante Schwankungen. Eine Trendanalyse der Veränderungen des Frauenanteils erwies sich für die Gesamtkategorie als signifikant (r^2^ = 0,585; *p* = 0,006). Darüber hinaus ergab eine Untersuchung einzelner Bereiche, dass die Veränderung im Anteil von 2013 bis 2024 für Plenarsitzungen (r^2^ = 0,707; *p* = 0,001) und Abstractsitzungen (r^2^ = 0,414; *p* = 0,033) signifikant war. Dennoch waren diese Trends gering und für Industriesymposien nicht signifikant (r^2^ = 0,110; *p* = 0,384) (Abb. 2a–d).

### Trendanalyse: Frauenanteil unter den Vorsitzenden (2013–2024)

Eine Trendanalyse der Veränderung des Frauenanteils unter den Vorsitzenden erwies sich für die Gesamtkategorie als signifikant (r^2^ = 0,777; *p* < 0,001). Auch die Betrachtung der einzelnen Bereiche ergab eine signifikante Veränderung des Frauenanteils von 2013 bis 2024 sowohl für Plenarsitzungen (r^2^ = 0,796; *p* < 0,001) als auch für Abstractsitzungen (r^2^ = 0,678; *p* = 0,002). Da von 2013 bis 2024 keine Frauen als Vorsitzende in Industriesymposien vertreten waren, blieb diese Analyse ergebnislos (Abb. 2a–d).

### Trendanalyse der Geschlechterverteilung bei den Vortragenden (2013–2024)

Die Trendanalyse der Geschlechterverteilung 2013 bis 2024 zeigte eine signifikante Veränderung (*p* < 0,001). Bis 2019 stellten Männer konstant mindestens 80 % der Vortragenden mit einem Maximum von 87 % im Jahr 2016. Erst 2024 überschritten Frauen erstmals die 30-Prozent-Marke und erreichten 33 %. Die Bereichsanalyse wies deutliche Unterschiede auf: In Plenarsitzungen zeigte sich ein signifikanter Anstieg weiblicher Vortragender (*p* < 0,001), obwohl Männer lange dominierten (Höchstwert 91 % im Jahr 2017). Ab 2021 lag der Frauenanteil durchgängig bei mindestens 20 % und erreichte 2023 und 2024 jeweils 29 %. Ein ähnlicher Befund zeigte sich für Abstractsitzungen (*p* = 0,003): Zwischen 2013 und 2023 waren mindestens 71 % der Vortragenden männlich (2013 und 2015: 76 %), während 2024 der Männeranteil auf 57 % sank. Frauen erreichten hier maximal 43 % (2024). In Industriesymposien zeigte sich kein Trend (*p* = NA); von 2015 bis 2024 traten lediglich 4 Frauen als Vortragende auf, während Männer durchgehend den Großteil ausmachten (88–100 %) (Tab. 1).

**Tab. 1:** Vortragende und Vorsitzende 2013 bis 2024 nach Geschlecht

	Vortragende			Vorsitzende		
	Männer	Frauen		Männer	Frauen	
Domäne	*n (%)*	*n (%)*	p*	*n (%)*	*n (%)*	p*
*Insgesamt*	–	–	*<* *0,001*	–	–	*<* *0,001*
2013	308 (84 %)	57 (16 %)	–	112 (93 %)	8 (7 %)	–
2014	277 (80 %)	68 (20 %)	–	100 (94 %)	6 (6 %)	–
2015	290 (83 %)	56 (17 %)	–	98 (92 %)	9 (8 %)	–
2016	208 (87 %)	32 (13 %)	–	106 (90 %)	12 (10 %)	–
2017	312 (85 %)	57 (15 %)	–	114 (90 %)	13 (10 %)	–
2018	193 (85 %)	35 (15 %)	–	88 (89 %)	11 (11 %)	^*‡*^*<* *0,001*
2019	219 (86 %)	36 (14 %)	–	50 (58 %)	36 (42 %)	
2021	163 (75 %)	55 (25 %)	–	41 (56 %)	32 (44 %)	–
2022	205 (77 %)	60 (23 %)	–	47 (56 %)	37 (44 %)	–
2023	183 (72 %)	71 (28 %)	–	60 (63 %)	35 (37 %)	–
2024	230 (67 %)	112 (33 %)	–	66 (59 %)	46 (41 %)	–
*Plenum*	–	–	*<* *0,001*	–	–	*<* *0,001*
2013	206 (89 %)	25 (11 %)	–	83 (95 %)	4 (5 %)	–
2014	157 (87 %)	24 (13 %)	–	65 (96 %)	3 (4 %)	–
2015	163 (89 %)	21 (11 %)	–	71 (91 %)	7 (9 %)	–
2016	175 (89 %)	21 (11 %)	–	96 (90 %)	11 (10 %)	–
2017	198 (91 %)	20 (9 %)	–	81 (89 %)	10 (11 %)	–
2018	124 (89 %)	16 (11 %)	–	72 (88 %)	10 (12 %)	^*‡*^*<* *0,001*
2019	171 (89 %)	22 (11 %)	–	42 (58 %)	31 (42 %)	
2021	104 (74 %)	36 (26 %)	–	32 (54 %)	27 (46 %)	–
2022	141 (80 %)	36 (20 %)	–	41 (60 %)	27 (40 %)	–
2023	141 (71 %)	57 (29 %)	–	48 (62 %)	30 (38 %)	–
2024	156 (71 %)	65 (29 %)	–	52 (59 %)	36 (41 %)	–
*Abstracts/Kurzvorträge*		–	*0,003*	–	–	*<* *0,001*
2013	102 (76 %)	32 (24 %)	–	29 (88 %)	4 (12 %)	–
2014	120 (73 %)	44 (27 %)	–	35 (92 %)	3 (8 %)	–
2015	122 (76 %)	35 (24 %)	–	26 (93 %)	2 (7 %)	–
2016	26 (72 %)	10 (28 %)	–	7 (88 %)	1 (12 %)	–
2017	110 (75 %)	37 (25 %)	–	29 (91 %)	3 (9 %)	–
2018	50 (75 %)	17 (25 %)	–	13 (93 %)	1 (7 %)	^‡^0,051
2019	38 (73 %)	14 (27 %)	–	5 (50 %)	5 (50 %)	
2021	47 (72 %)	18 (28 %)	–	6 (55 %)	5 (45 %)	–
2022	60 (71 %)	24 (29 %)	–	5 (33 %)	10 (67 %)	–
2023	37 (73 %)	14 (27 %)	–	7 (58 %)	5 (42 %)	–
2024	63 (57 %)	47 (43 %)	–	11 (52 %)	10 (48 %)	–
*Industriesymposien*		–	NA	–	–	NA
2013	NA	NA	–	NA	NA	–
2014	NA	NA	–	NA	NA	–
2015	5 (100 %)	0	–	1 (100 %)	0	–
2016	7 (88 %)	1 (12 %)	–	3 (100 %)	0	–
2017	4 (100 %)	0	–	4 (100 %)	0	–
2018	19 (90 %)	2 (10 %)	–	3 (100 %)	0	^‡^0,999
2019	10 (100 %)	0	–	3 (100 %)	0	
2021	12 (92 %)	1 (8 %)	–	3 (100 %)	0	–
2022	4 (100 %)	0	–	1 (100 %)	0	–
2023	5 (100 %)	0	–	5 (100 %)	0	–
2024	11 (100 %)	0	–	3 (100 %)	0	–

*NA* „not applicable“ (nicht zutreffend)

* Cochran-Armitage-Test für Trends

^‡^ Exakter Test nach Fisher

### Trendanalyse der Geschlechterverteilung bei den Vorsitzenden (2013–2024)

Die Analyse der Gesamtzahl der Vorsitzenden zeigte von 2013 bis 2024 einen deutlichen Trend (*p* < 0,001). Bis 2018 stellten Männer konstant mindestens 89 % der Vorsitzenden mit einem Maximum von 94 % im Jahr 2014. Ab 2019 überschritten Frauen erstmals die 40-Prozent-Marke (42 %) und erreichten 2021 und 2022 ihren Höchstwert von 44 %. Eine differenzierte Betrachtung der einzelnen Bereiche verdeutlichte klare Unterschiede. In den Plenarsitzungen dominierten Männer über den gesamten Zeitraum (54–96 %; Frauen 4–46 %), wobei ein signifikanter Trend vorlag (*p* < 0,001). Zwischen 2013 und 2018 stellten sie mindestens 88 % der Vorsitzenden mit einem Spitzenwert von 96 % im Jahr 2014. Ab 2019 zeigte sich eine deutliche Verschiebung, die 2021 in einem Frauenanteil von 46 % gipfelte (*p* < 0,001). Auch für Abstractsitzungen ergab sich ein signifikanter Trend (*p* < 0,001). Von 2013 bis 2018 waren mindestens 88 % der Vorsitzenden männlich (Höchststand 93 % in 2015 und 2018). Ab 2019 wurde erstmals Parität erreicht (50/50), und 2022 lag der Frauenanteil bei 67 %, dem höchsten Wert im gesamten Zeitraum. In Industriesymposien hingegen waren über alle Jahre ausschließlich Männer als Vorsitzende vertreten (100 %), sodass keine Trendanalyse möglich war (*p* = NA) (Tab. 1).

### Auswirkungen des Beschlusses zur paritätischen Besetzung der Vorsitze

Unter Berücksichtigung des Beschlusses zur paritätischen Besetzung der Vorsitze des DGVS-Vorstands aus dem Jahr 2019 ist ein deutlicher Anstieg des Gesamtanteils weiblicher Vorsitzenden in den viszeralchirurgischen Sitzungen zu beobachten, der von 11 % im Jahr 2018 auf 42 % im Jahr 2019 gestiegen ist (*p* < 0,001). Für eine differenzierte Analyse wurde ein Vergleich der Geschlechterverteilung vor und nach der Umsetzung des Beschlusses durchgeführt. Die Veränderung der Geschlechterverteilung der Vorsitzenden in den Plenarsitzungen zur Viszeralchirurgie war signifikant (*p* < 0,001). Ein Trend war jedoch nicht unter den Industriesymposien (*p* = 0,999) zu sehen (Tab. 1).

### Dynamik der Geschlechterparität – Anteil weiblicher Vortragender und Vorsitzender im Zeitverlauf

**Abb. 3:**
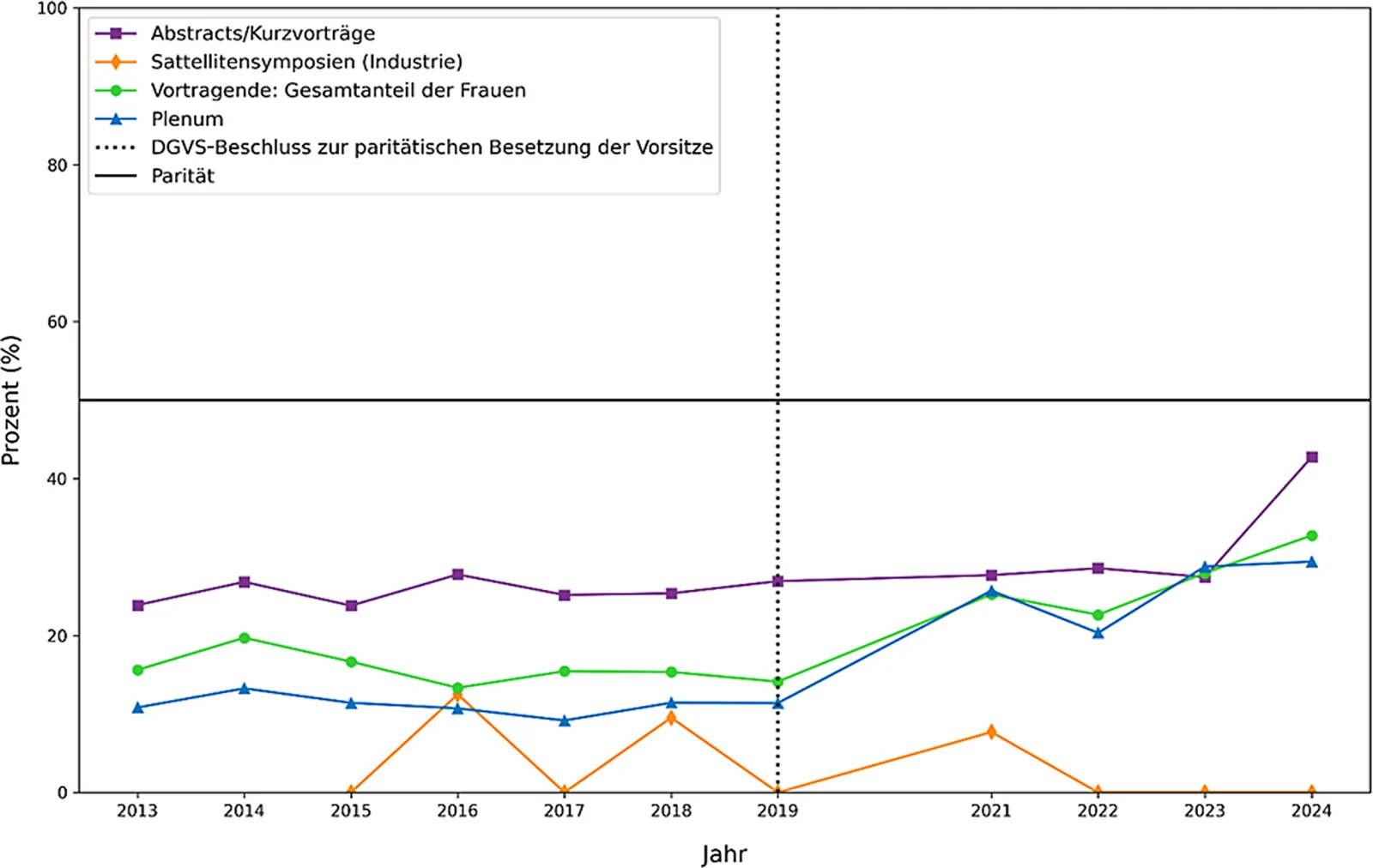
Entwicklung des prozentualen Anteils von Frauen unter den Vortragenden von 2013 bis 2024, ohne 2020. *DGVS* Deutsche Gesellschaft für Gastroenterologie, Verdauungs- und Stoffwechselkrankheiten

Insgesamt erreicht der Anteil weiblicher Vortragender in den viszeralchirurgischen Sitzungen des Deutschen Kongresses für Viszeralmedizin im Zeitraum von 2013 bis 2024 kein einziges Mal die Parität. Im Allgemeinen ist der Anteil von Frauen in den Abstractsitzungen am höchsten. Im Gegensatz dazu weisen die Industriesymposien durchweg den niedrigsten Anteil weiblicher Vertreterinnen auf mit Ausnahme der Daten aus dem Jahr 2016. Hier liegt der Frauenanteil bei Industriesymposien höher als der in Plenarsitzungen (Abb. 3).

**Abb. 4:**
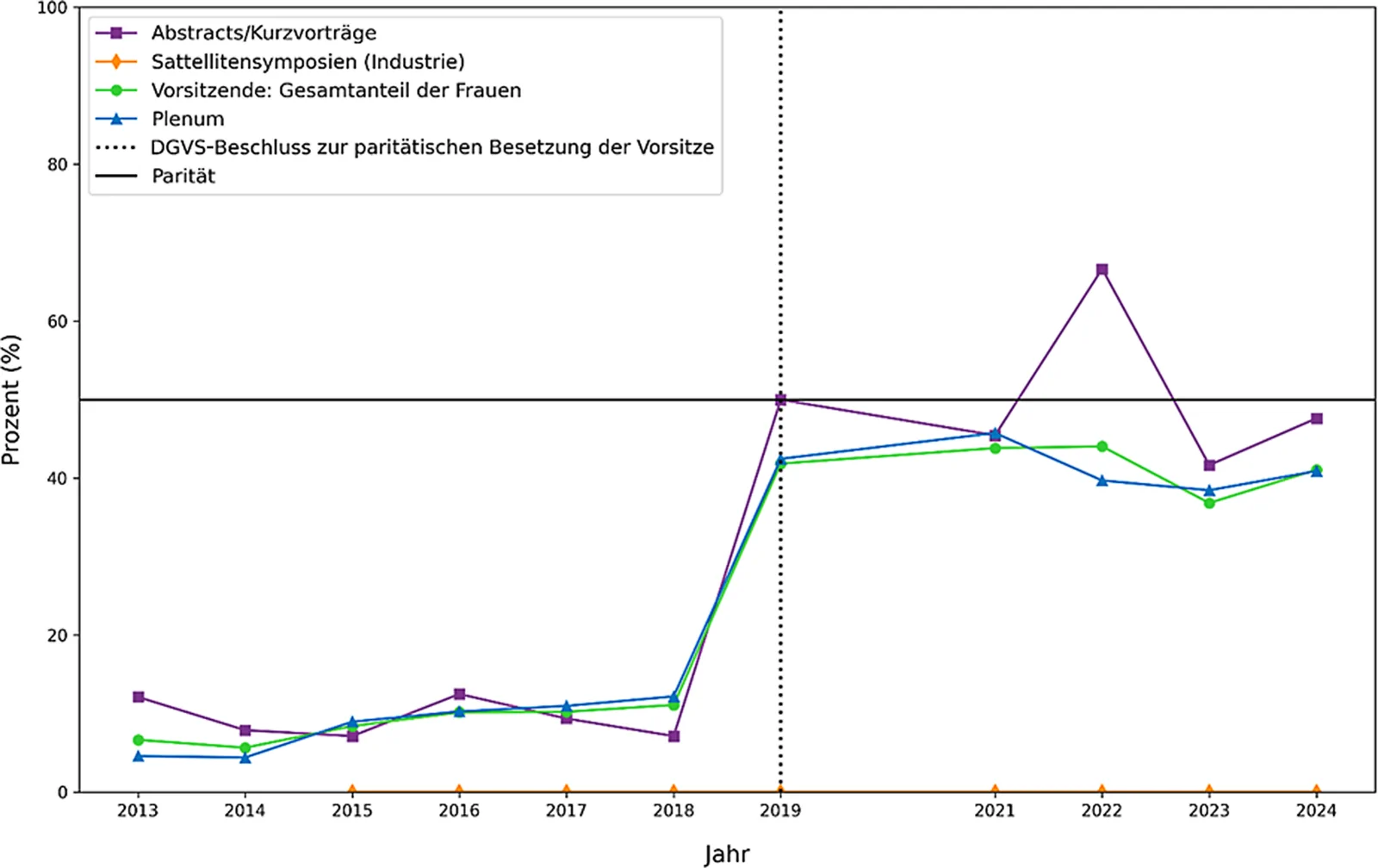
Entwicklung des prozentualen Anteils von Frauen unter den Vorsitzenden von 2013 bis 2024, ohne 2020. *DGVS* Deutsche Gesellschaft für Gastroenterologie, Verdauungs- und Stoffwechselkrankheiten

Insgesamt zeigt der Anteil weiblicher Vorsitzender in den viszeralchirurgischen Sitzungen des Deutschen Kongresses für Viszeralmedizin einen Aufwärtstrend in Richtung Parität. Bemerkenswert ist der Anteil weiblicher Vorsitzender in den Abstractsitzungen im Jahr 2019 mit 50 % und im Jahr 2022 mit 67 % (Abb. 4).

## Kongresspräsidentschaften und Auszeichnungen

**Abb. 5:**
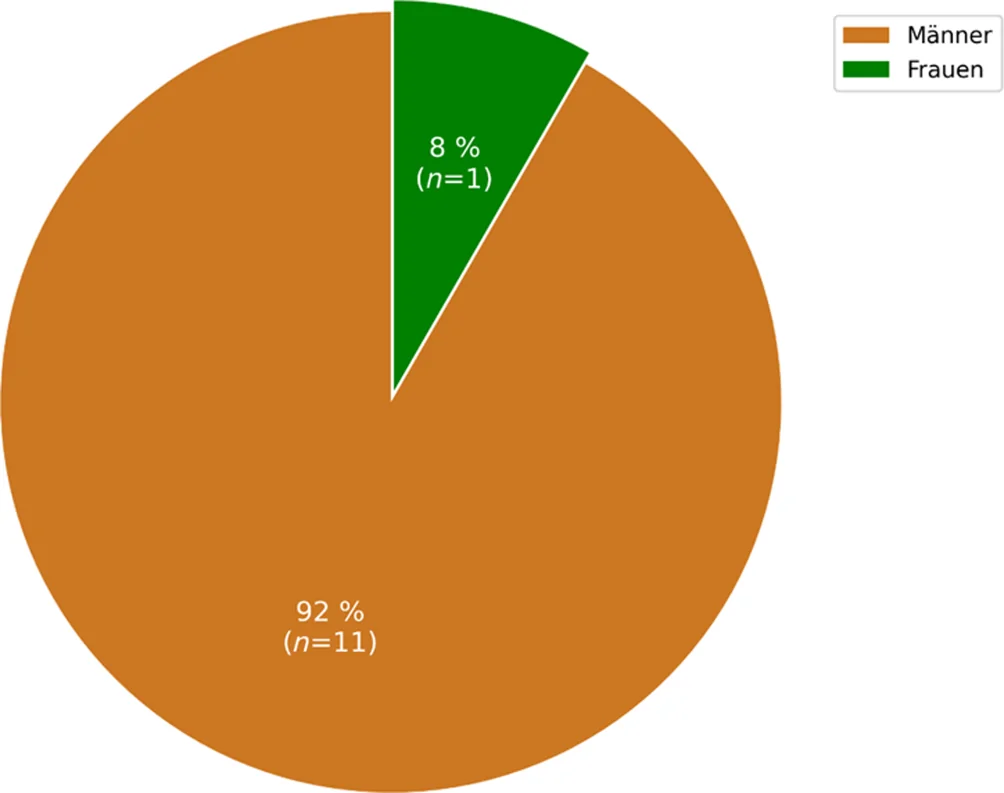
Prozentualer Anteil von Frauen unter den Kongresspräsidentschaften von 2013 bis 2024

Von 2013 bis 2024 war die Position der viszeralchirurgischen Kongresspräsident*innen überwiegend männlich besetzt (92 %). Das Jahr 2021 stellte diesbezüglich eine Besonderheit dar, da erstmals eine Frau die Kongresspräsidentschaft der DGAV übernahm (Abb. 5).

**Abb. 6:**
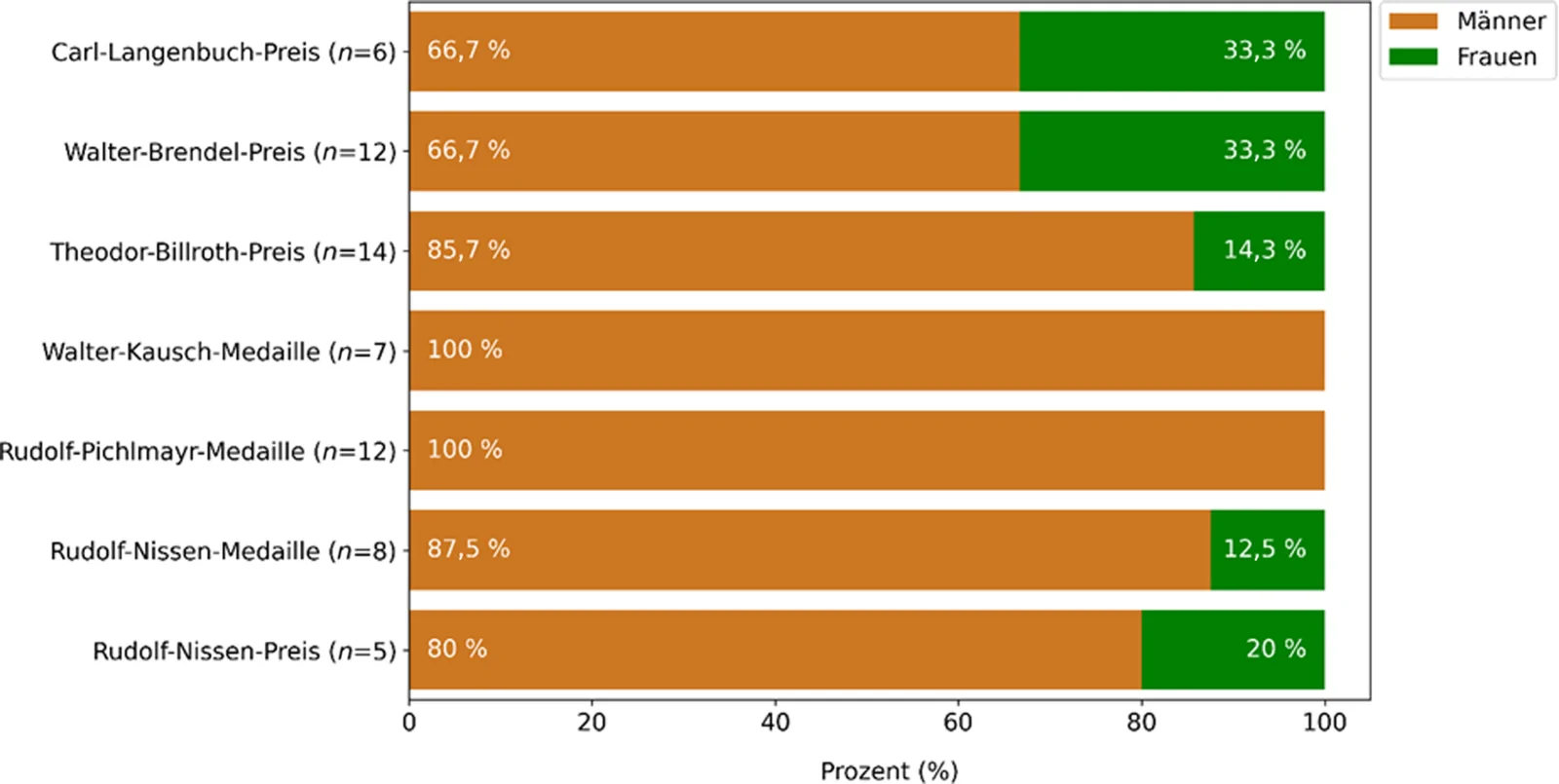
Prozentualer Anteil der Preisträger nach Geschlecht von 2013 bis 2024

Die Daten zu Auszeichnungen und Medaillen zeigen eine anhaltende Ungleichheit, wobei Männer in diesen Bereichen durchweg dominieren. So wurden beispielsweise 100 % der renommierten Walter-Kausch-Medaille und 100 % der Rudolf-Pichlmayr-Medaille ausschließlich an Männer verliehen. Ebenso wurden die Rudolf-Nissen-Medaille und der Theodor-Billroth-Preis überwiegend an Männer vergeben (88 % bzw. 86 %). Die Träger des Carl-Langenbuch-Preises und des Walter-Brendel-Preises waren jeweils zu 67 % männlich (Abb. 6).

## Diskussion

Die vorliegende Analyse der weiblichen Repräsentanz auf dem deutschen Viszeralmedizin-Kongress zwischen den Jahren 2013 und 2024 zeigt eine deutliche Unterrepräsentation von Frauen in allen viszeralchirurgischen Bereichen. Der 2019 eingeführte Beschluss zur paritätischen Besetzung der Vorsitze führte zwar zu einem signifikanten Anstieg weiblicher Vortragender und Vorsitzender, erreichte jedoch keine vollständige Gleichverteilung. Parität wurde lediglich in den Abstractsitzungen erzielt, bedingt durch einen Rückgang männlicher Vorsitzender, während Präsidentschaften, Auszeichnungen, Industriesymposien und Plenarvorträge mit Frauenanteilen von nur 0–33 % weiterhin besonders geringe Beteiligung aufwiesen.

Der durchschnittliche Frauenanteil unter Vortragenden betrug 20 % und lag damit deutlich unter den Werten internationaler Studien [1, 3, 15]. Trotz eines moderaten Anstiegs in Plenar- und Abstractsitzungen blieben die Maximalwerte mit 29 % bzw. 43 % begrenzt. Die Zunahme ab 2021 lässt sich vermutlich auf den Paritätsbeschluss der DGVS sowie die erstmals weibliche Kongresspräsidentschaft zurückführen [24]. Internationale Untersuchungen zeigen ähnliche strukturelle Muster: Arora et al. berichten von 30 % weiblichen Vortragenden bei gleichzeitig 36,6 % Sitzungen ohne Frauen [1]. In der Chirurgie liegen die Frauenanteile zwar teilweise höher (USA: 22 %, Großbritannien: 22 %), aber dennoch über denen der deutschen Plenarsitzungen (Ø 16 %) [15, 21], was angesichts der karriererelevanten Bedeutung dieser Formate problematisch ist [22, 23]. Die Abstractsitzungen erreichten mit 28 % den höchsten Frauenanteil, was über den Werten anderer chirurgischer Fachgebiete liegt (kardio-/thoraxchirurgisch: 15 %) [14], jedoch v. a. die stärkere Präsenz von Frauen in transparenten Bewerbungsverfahren widerspiegelt.

In Industriesymposien blieb die weibliche Beteiligung besonders niedrig: In 6 von 9 Jahren lag sie bei 0 % mit einem Maximum von lediglich 12 %. Auch international zeigt sich Unterrepräsentation, wenn auch teilweise auf höherem Niveau (18 %) [13]. Gastroenterologische Symposien desselben Kongresses wie dem hier untersuchten weisen deutlich höhere Frauenanteile (15–33 %) auf [16]. Da Industriekooperationen entscheidend für Forschungsfinanzierung und Sichtbarkeit sind, führt die geringe weibliche Präsenz in diesen Formaten zu klaren Nachteilen hinsichtlich Fördermitteln und Karrierechancen; der Mangel an Transparenz in Auswahlprozessen verstärkt dies [27].

Zusammenfassend bleibt auf dem Viszeralmedizin-Kongress im untersuchten Zeitraum der Anteil der Rednerinnen über alle untersuchten Kategorien hinweg unter dem Anteil von Frauen in der Allgemein- und Viszeralchirurgie, der 2024 bei knapp 36 % lag, und ist damit nicht repräsentativ. Das Ziel einer proportionalen Repräsentation von Frauen hingegen birgt die Gefahr, den Status quo der Chirurgie als männlich dominiertes Fach zu festigen.

Auch bei Vorsitzfunktionen dominierten Männer deutlich. In Plenarsitzungen lag der Frauenanteil zwischen 4 % und 46 %, in Industriesymposien durchgängig bei 0 %, während nur die Abstractsitzungen 2024 einen Frauenanteil von 67 % erreichten. Internationale Vergleiche (USA: 24 %, Europa: 10–22 %) bestätigen diese Muster [14, 23]. Vorsitzfunktionen sind wichtige Karrierebausteine, und die Ergebnisse unterstreichen in Übereinstimmung mit aktueller Literatur, dass die angewandten verpflichtenden Gleichstellungsmaßnahmen wirksam sind [2, 23].

Bei den 7 vergebenen Preisen, deren Vergabe ausschließlich über Nominierungen erfolgt [10], lag der Frauenanteil zwischen 0 % und 33 %, 2 Auszeichnungen gingen im gesamten Untersuchungszeitraum nie an eine Frau. Eine umfassende Analyse über 163 medizinische Fachgesellschaften zeigt ebenfalls eine geringe weibliche Beteiligung an prestigeträchtigen Ehrenpreisen (29 %) [17]. Fehlende Transparenz der Nominierungskriterien lässt dabei mögliche strukturelle Benachteiligung vermuten [23, 27].

Auch in den Präsidentschaften waren Frauen deutlich unterrepräsentiert: Zwischen 2013 und 2024 waren 92 % der Kongresspräsident*innen männlich. Erst 2021 wurde eine Viszeralchirurgin in dieses Amt berufen, was von einem Anstieg weiblicher Vortragender begleitet war. Die Literatur zeigt konsistent, dass weibliche Führungspersonen die Beteiligung von Frauen in wissenschaftlichen Programmen erhöhen [4, 15] und als Vorbilder für Karrierewege und erfolgreiche Facharztweiterbildungen essenziell sind [13, 20]. Ihr Mangel verstärkt bestehende Ungleichheiten sowie den Nachwuchsmangel in der Chirurgie [7, 23].

Die Unterrepräsentation von Frauen in prestigeträchtigen Funktionen auf dem Viszeralmedizin-Kongress ist ein stellvertretendes Beispiel für die Ungleichheit der Karrierewege von Chirurginnen und Chirurgen und nicht in Einklang zu bringen mit der Annahme, dass klinische und wissenschaftliche Talente in beiden Geschlechtern gleichermaßen vertreten sind. Wie die vorliegende Analyse und vergleichbare internationale Studien zeigen, werden vergleichbare Leistungen von Frauen und Männern systematisch aufgrund struktureller Verzerrungen unterschiedlich wahrgenommen, bewertet und sichtbar gemacht [5, 12, 19, 31, 35]. Eine paritätische Verteilung relevanter Positionen auf Fachkongressen stellt eine effektive Option dar, diese Dysbalancen auf dem Karriereweg von Frauen zu ebnen.

Die vorliegende Analyse darf als Anstoß verstanden werden, Repräsentation und Sichtbarkeit zu diskutieren und neu zu denken.

## Limitationen

Die Aussagekraft der Studie muss vor dem Hintergrund einiger Limitationen betrachtet werden. Namen erlauben keine zuverlässige Bestimmung der Geschlechtsidentität. Die Analyse erfasst daher nicht die Geschlechtsidentität der Personen. Zudem kann die Annahme eines binären Geschlechts die Geschlechtsidentität falsch abbilden. Weitere potenzielle Limitationen sind:

- Intransparenz der Teilnahmeeinladungen und Nominierungen für Auszeichnungen,
- fehlende Daten zur Geschlechterverteilung der abgelehnten Einladungen.

## Fazit für die Praxis

- Insgesamt waren Frauen in der Viszeralchirurgie in der letzten Dekade in allen untersuchten Bereichen des deutschen Viszeralmedizin-Kongresses unterrepräsentiert.
- Nur in den Vorsitzen der Abstractsitzungen überschritt der Frauenanteil die Parität.
- In den hoch angesehenen und für den beruflichen Aufstieg relevanten Kategorien (Präsidentschaft, Auszeichnungen, Vortragende in Plenarsitzungen, Industriesymposien) lag der Frauenanteil maximal bei 33 %, wobei mehrfach ein Männeranteil von 100 % gezeigt werden konnte.
- Initiativen zur paritätischen Besetzung zeigen einen signifikanten Trend zur Steigerung des Frauenanteils und stellen ein vielversprechendes Mittel zur Erlangung von Chancengleichheit dar.
- Ein transparentes Verfahren zur Besetzung sowohl führender als auch durch die Industrie besetzter Positionen und Preisträgerschaften auf dem deutschen Viszeralmedizin-Kongress ist dringend erforderlich.

## Data Availability

Die erhobenen Datensätze können auf begründete Anfrage in anonymisierter Form beim korrespondierenden Autor angefordert werden. Die Daten befinden sich auf einem Datenspeicher an der Medizinischen Hochschule Hannover.
